# Interfacial interaction study of EDTA with the defect structure of Fe_3−*δ*_O_4_ passive film in an aggressive alkaline medium based on the lattice theory of point defects

**DOI:** 10.1039/d1ra07171h

**Published:** 2022-01-26

**Authors:** I. Azamian, S. R. Allahkaram, M. Johari, F. Teymouri

**Affiliations:** School of Metallurgy and Materials Engineering, College of Engineering, University of Tehran Iran Akaram@ut.ac.ir

## Abstract

Despite extensive research on the matter of corrosion inhibition efficiency, the interactions between the defect structure of the passive layer and the inhibitor molecules still remain poorly understood. In this study, the corrosion inhibition mechanism of ethylenediamine-tetraacetic acid as a carboxylate-based organic inhibitor on steel specimens in simulated concrete pore solution was studied. The point defect model was used to describe the response of the passive oxide film on the steel surface to the perturbation caused by the addition of the carboxylate compound. The electrochemical behavior of the steel specimens was evaluated through open circuit potential, electrochemical impedance spectroscopy and potentiodynamic analysis. The reduction in efficiency outside the optimal concentrations was discussed from an electrochemical point of view. We suggest that the performance of the inhibitor is highly dependent on the positively charged entities on the passive layer including anion vacancies and interstitial cations. To further investigate the physicochemical behavior of the organic molecules, density functional theory and adsorption isotherms were applied. The topography and morphology of the surface were analyzed through scanning electron microscopy. To confirm the inhibitive effect of EDTA, the elements and chemical bonds present on the surface were characterized *via* X-ray photoelectron spectroscopy. The surface analysis confirmed that the addition of EDTA formed a network of chemical bonds, which significantly hindered the corrosion phenomenon.

## Abstract 2

Despite extensive research on the matter of corrosion inhibition efficiency, the interactions between the defect structure of the passive layer and the inhibitor molecules still remain poorly understood.

## Graphical abstract

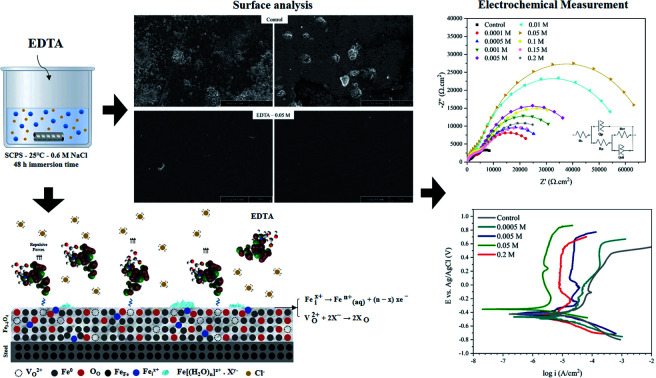

## 1. Introduction

Considering recent progress in building material selection and additive development, it is safe to say that concrete materials are well studied. However, in most studies, the primary focus has been the mechanical characteristics of the structures and only under static conditions. Nevertheless, the problem of aggressive agents attacking the steel rebar and causing corrosion is generally observed, which leads to material degradation and subsequent mechanical failure of the structure.

Usually, the highly alkaline condition of concrete ensures a strong passive state, which is a protective anti-corrosion oxide/hydroxide layer on the steel bar. However, when it becomes contaminated with aggressive agents such as chloride ions or the concrete undergoes carbonation processes, the stability of this layer starts to decrease and pitting corrosion starts when the chloride content exceeds a critical amount of 0.4–1% by cement weight.^1^ Simulated concrete pore solution (SCPS) has been used to investigate the factors affecting rebar corrosion under diverse conditions, and it is chosen as the evaluation method in the present study.

Among the approaches for controlling the negative effects of these occurrences, corrosion inhibitors have drawn much attention in the past decades. In the case of steel-reinforced concrete, certain inorganic inhibitors such as nitrites are used in industries to control corrosion. However, they are usually associated with the risk of toxicity and efficiency reduction in long-term applications.^2^ Organic inhibitors, especially compounds containing carboxylic groups, have been reported to be effective inhibitors of passive film breakdown. Ormelles *et al.*^3^ studied different organic inhibitors in SCPS using the cyclic polarization method. They found that carboxylate substances show outstanding inhibition effectiveness, making them the most promising candidates among organic compounds.

Although several studies on steel passivity in the presence of organic inhibitors in alkaline media have been carried out, the understanding of the inhibition mechanism, surface/electrolyte interactions, and actual physicochemical processes near the surface is still uncertain. The primary presumption is that in the process of inhibition, the chemical groups on the organic compounds are adsorbed on the surface and form a semi-stable coverage against aggressive agents, which may slow down both the cathodic and anodic reactions and lessen the corrosion process in the steel reinforcement.^4,5^ Another assumption is that these compounds have the ability to act as chelating agents, with the vacant d-orbital of iron metal and the lone pair of electrons of their heteroatoms forming a covalent bond.^6^

Bing *et al.*^7^ employed electrochemical measurement and quantum chemical analysis to investigate the relationship between the corrosion inhibition efficiency and different alkylene chain lengths of carboxylate inhibitors. They reported that the adsorption capacity of the inhibitors increased with an increase in the distance between the C=C bond and COO^−^ group. They also showed that with an increase in the alkylene chain length, the absolute surface charge value increases, therefore enhancing the surface adsorption of organic molecules. The relation between the inhibition action and number of carboxylate groups was observed in another study.^3^ It was found that the efficiency decreases drastically in organic molecules with more than four carboxylate groups. However, the influence of the inhibitor concentration was not accurately examined in that experiment.

Ethylenediamine-tetraacetic acid (EDTA) is one of the earliest compounds that was utilized as an organic inhibitor in SCPS and was used in the present study. It has two amine groups on the middle part of its alkylene chain and two carboxylate groups on each end. Macdonald *et al.*^8,9^ investigated the effect of EDTA as a chelating agent on the passivity of iron in a borate buffer solution. Utilizing the point defect model (PDM), they reported that the interactions on the metal/electrolyte interface occur through the vacancy defects on the imperfect surface layer. Szklarska *et al.*^10^ performed periodic passivation reduction cycles on a metal immersed in a solution with a pH value of 12 in the presence of EDTA and reported that this compound did not affect the formation of the passive film but drastically enhanced the cathodic reduction of the layer.

Also, there have been many studies on the subject of organic inhibitors, but to date, there is no proper explanation for their actual inhibition mechanism. Besides, most of the studies failed to accurately simulate the real situation occurring on the surface and in the electrolyte bulk. For example, in most of the works, the immersion time for the metal is 1 hour or less, but it cannot be guaranteed that a passive layer develops within a period of less than 48 hours, especially when the chemical composition of the electrolyte is just Ca(OH)_2_ and does not consist of NaOH or KOH, which are necessary for concrete pore simulation.^11–13^ Furthermore, in some works, aggressive ions are added after a certain exposure time to ensure a strong passive state before corrosion. However, this is not consistent with reality given that agents such as atmospheric chloride are always present.

The present study aimed to assess the action mechanism of EDTA as an organic corrosion inhibitor in an SCPS in the presence of chloride ions. Firstly, the inhibition effectiveness in different concentrations was studied *via* open circuit potential (OCP) measurement and electrochemical impedance spectroscopy (EIS). The polarization method was also used to investigate the stability of the modified passive film against chloride-induced localized corrosion. The morphology of the metal surface was investigated *via* field-emission scanning electron microscopy (FE-SEM). X-ray photoelectron spectroscopy (XPS) was also utilized to investigate the surface composition. In parallel, to study the chemical characteristics and anticorrosive capability of EDTA, quantum chemical calculations were performed. Finally, based on the results, to achieve a comprehensive understanding of the physicochemical processes and inhibition mechanism, the point defect model was exploited.

## 2. Experimental

### 2.1. Electrochemical measurement

All experiments were carried out in a typical three-electrode electrochemical cell in pH 13 SCPS, which was a saturated Ca(OH)_2_ solution containing KOH 0.05 M and NaOH 0.05 M with various amounts of EDTA, at room temperature after 48 hours of exposure. The concentration of the added compound was 0.0001 M, 0.0005 M, 0.001 M, 0.005 M, 0.01 M, 0.05 M, 0.1 M, 0.15 M, and 0.2 M, which was chosen based on the observation that intermediate inhibition efficiencies (20–90%) are attained in this particular range. Analytical grade chemicals were purchased from Merck Co. A Q235 carbon steel specimen was used as the working electrode. Before applying the solution to the metal, the specimens were abraded with emery paper from 80 to 1500 grade, and then washed with distilled water and acetone. All data was normalized to the area of the exposed surface of the working electrode, which was 1.0 cm^2^. Platinum and Ag/AgCl were used as the counter and the reference electrodes, respectively. EIS and potentiodynamic polarization scans were performed after the OCP reached the steady state. Prior to each experiment, the OCP trend was measured for 48 hours of immersion. An AUTOLAB PGSTAT 302N instrument was used for electrochemical analysis. For impedance analysis, the measurements were performed in the range of 100 kHz to 10 MHz. In the potentiodynamic polarization measurements, the polarization potential was scanned from 0.8 to +1.2 V *vs.* OCP, with a scan rate of 10 mV min^−1^. The pH of the solution was also monitored to investigate the effect of long-term exposure in electrolyte for a period of 6 months.

### 2.2. Adsorption isotherms

The EIS parameters were used to investigate the adsorption behaviour of the inhibitor. The adsorption of a substance is usually described through isotherms, that is, the amount of adsorbate on the adsorbent as a function of concentration at a constant temperature. The isotherms provide information on the interaction between the metal surface and inhibitors. The Langmuir, generalized Langmuir–Freundlich, and Temkin adsorption isotherms were used in this study, which are represented by eqn (1)–(3), respectively,^14–16^1
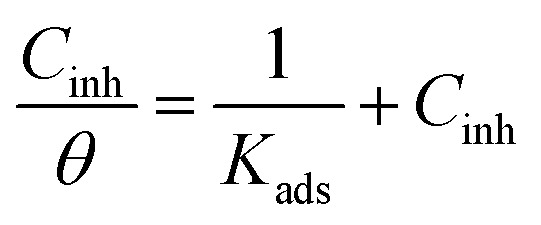
2
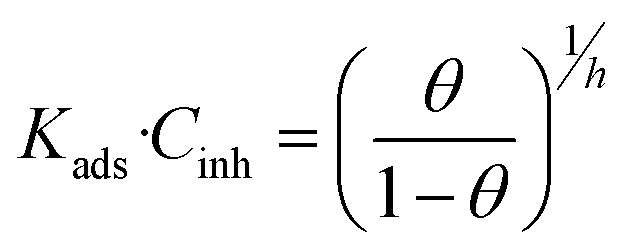

3 *K*_ads_·*C*_inh_ = e^*fθ*^

where *C*_inh_ is the inhibitor concentration, *K*_ads_ is the equilibrium constant of the adsorption/desorption reaction, *f* is the molecular interaction, and *h* is the heterogeneity parameter, corresponding to the adsorption energy distribution on the surface. *θ* is the fractional surface coverage by inhibitor, which is calculated based on the assumption that there is a relationship between charge transfer resistance and the corrosion rate. For this purpose, the exported data from EIS technique were used to examine the surface coverage of the inhibitor molecules based on eqn (10), which is defined in Section 3.2.

### 2.3. Surface analysis

The surface morphologies and compositions of the metal samples in the SCPS in the absence and presence of the inhibitor were analyzed *via* FE-SEM examinations, which were performed using a MIRATESCAN-IROST scanning electron microscope. To investigate the adsorption mechanism on the defective structure of the passive layer, XPS measurement was carried out employing a BesTec instrument and K-Alpha system. The values obtained for the binding energy were calibrated according to the C 1s peak at 284.8 eV. Based on the chemical composition of EDTA, the high-resolution spectra of Fe 2p, C 1s, O 1s, Cl 2p and N 1s were measured. The samples used for surface analysis were obtained under the same condition as that for the electrochemical measurements.

### 2.4. Computer simulations

To further investigate the obtained results and scrutinize the relationship between the experimental data and the molecular electronic properties of EDTA, quantum chemical calculations were performed to predict the adsorption and inhibition phenomenon of carboxylate molecules. Geometrical optimizations and frequency calculations were carried out employing a density functional theory (DFT) method using the 6-311G basis set. The molecule was built using Gauss View, 5.0 implemented in the Gaussian 09 package and the optimization was executed without any geometry constraints. The B3LYP functional was used for calculation of all the optimized structures such as *E*_HOMO_ and *E*_LUMO_.

To analyze the role of the molecular structure of EDTA and its electronic properties on both the inhibition performance and the efficiency drop in critical parameters, its HOMO and LUMO energy values, and their related parameters, ionization potential, *I*, and electron affinity, *A*, were calculated using following relations:^17^

4 *I* = −*E*_HOMO_

5 *A* = −*E*_LUMO_

The energy gap (Δ*E* = *E*_LUMO_ − *E*_HOMO_), as a function of the reactivity of the inhibitor molecule towards adsorption on the metallic surface and dipole moment, which arises from differences in electronegativity, were also measured through DFT calculations. In this study, for the purpose of comparison, tetracyanoethylene (TCNE) was chosen as a ref. 18. Other quantum parameters such as electronegativity, *χ*, global hardness, *η*, chemical softness, *S*, and electron-donating ability were calculated using eqn (6)–(9), respectively,^19^ as follows:6
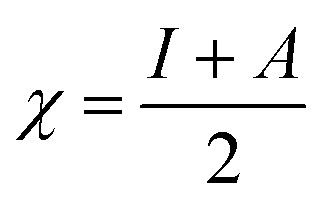
7
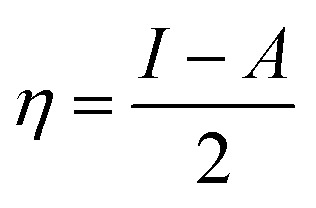
8
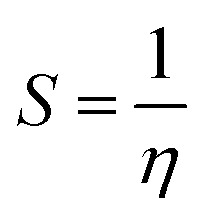
9
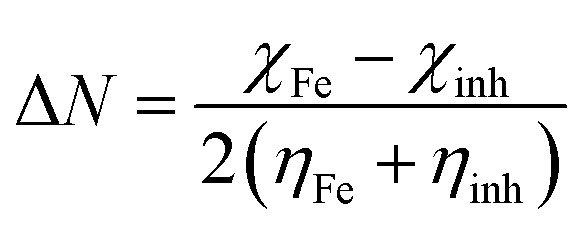
where the theoretical value for *χ*_Fe_ is 7 eV mol^−1^ and value of *η*_Fe_ = 0 eV mol^−1^ for steel was considered.

## 3. Results and discussion

### 3.1. Open circuit potential

**Fig. 1:**
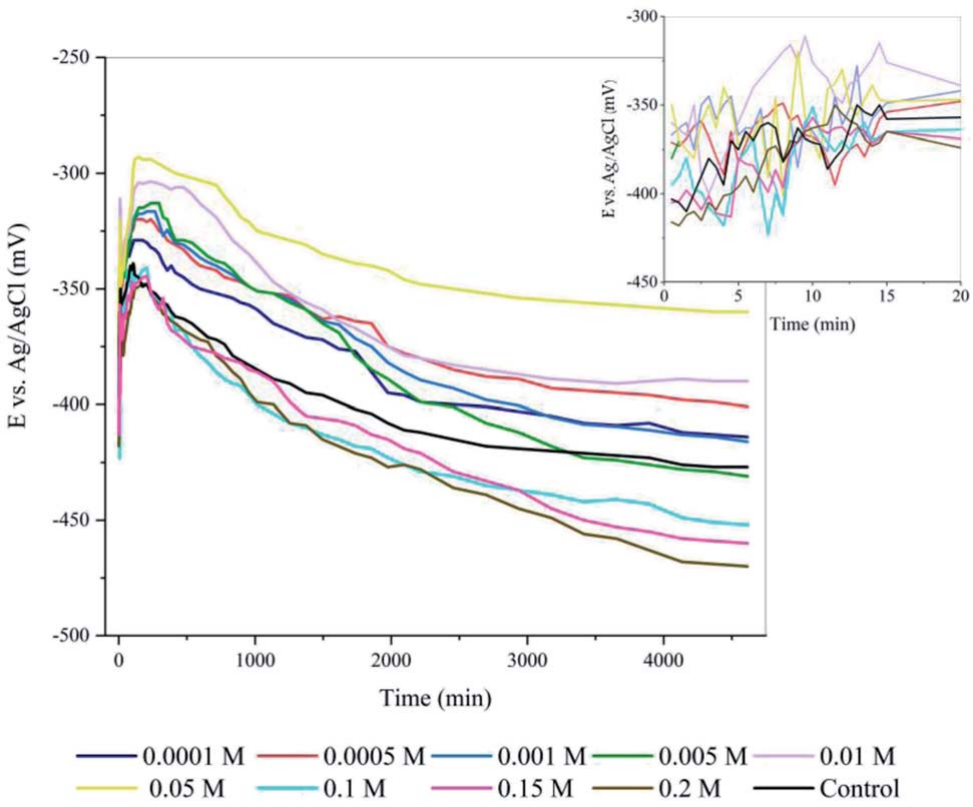
Open circuit potential values for steel sample exposed to SCPS contaminated with 0.6 M NaCl after 48 hours of immersion in the presence of different concentrations of EDTA.

Fig. 1 shows the time-dependent changes in the free corrosion potential of the metal samples in the presence of EDTA. The presence of an alkaline environment resulted in the formation of an Fe_3−*δ*_O_4_ (0 < *δ* < 0.33) passive film on the surface. It is well known that the point of zero charge (PZC) in hydrous oxides of carbon steel happens in the pH range of 8–9.^11,15,20^ Thus, at the pH value of 13 in the testing environment, the electrode surface is anticipated to be negatively charged. This is mainly because of the presence of excessive OH^−^ groups in the solution. Hence, other negatively charged species such as chloride ions and carboxylate groups have to compete with each other and also with OH^−^ to reach the surface.^3,5,21,22^ This notion is dependent on various factors and especially the concentration of the species involved in the system. For example, in an inhibitor-free alkaline environment, it has been proven that the passive film and OH^−^ adsorption layer can offer great protection against Cl^−^. Alternatively, it is a well-known fact that the protection can be guaranteed only if the [Cl^−^]/[OH^−^] ratio is less than 0.6,^23^ meaning that at a certain point, the competitive adsorption favors Cl^−^. The other factors regarding this issue are discussed in the EIS and quantum calculation sections.

The reason for the potential fluctuations in the first 15 min of immersion is the competitive process of adsorption between the chloride and organic groups. As illustrated in Fig. 1, for contents less than 0.05 M, the free corrosion potential in the presence of EDTA shifted to more positive values, and after approximately 30 hours, reached a steady-state. This result reflects the lower activity of the surface, indicating that the occurrence of physicochemical processes, including chelation, adsorption, and chemical reactions, is less active. However, with the further addition of EDTA, the potential decreased drastically, which offers a more active surface engaged in more interactions. At concentrations of 0.05 M and 0.2 M, the highest and the lowest steady-state potential of −360 mV and −528 mV, were achieved, respectively.

### 3.2. EIS analysis

**Fig. 2:**
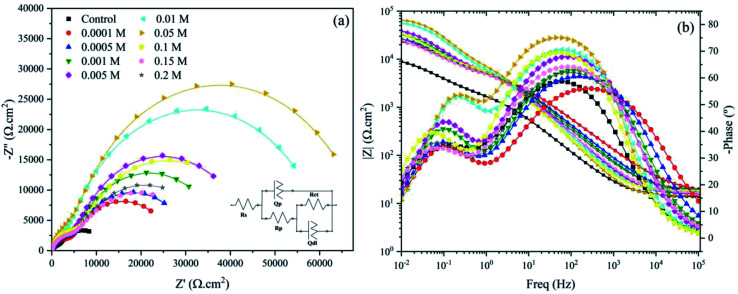
Electrochemical equivalent circuit and Nyquist (a) and Bode (b) plots obtained for C-steel corrosion in SCPS contaminated with 0.6 M NaCl containing different concentrations of EDTA after 48 hours immersion at 25 °C.

Fig. 2 shows the impedance spectra obtained for the steel samples in the presence of different contents of the inhibitor. The EIS results were simulated using [*R*_s_(*Q*_p_[*R*_p_(*R*_ct_*Q*_dl_)])] as the equivalent circuit, which is schematically shown in Fig. 2a. The experimental data was fitted using the Zview software, and the outcomes are shown in Table 1. Here *R*_s_ is the resistance of the solution. *R*_ct_ and *Q*_ct_ represent the charge transfer resistance at the interface and the double layer (DL) capacitance, corresponding to the processes occurring at high frequencies, while *R*_p_ and *Q*_p_ are the resistance and capacitance of the passive layer, which are related to the processes occurring in the passive film at low frequencies. The constant phase element *Q* was introduced instead of a capacitor to describe the non-ideal behaviour of the system due to the presence of physical, chemical, and geometrical heterogeneities. According to the characteristics of the obtained plots, a two-times constant circuit was selected, which is usually practical when at least two different interfaces with different interactions are involved in the system. In this case, the low-frequency time-constant can be related to the charge transfer at the interface of steel and film, and the high-frequency time-constant can be attributed to the capacitive behaviour of the passive layer.

**Table 1:** EIS parameters for different concentrations of EDTA in SCPS contaminated with 0.6 M NaCl

Sample	*R*_s_ (Ω cm^−2^)	*Q*_p_ (μs^*n*^ Ω^−1^ cm^−2^)	*n*	*R*_p_ (Ω cm^−2^)	*Q*_dl_ (μs^*n*^ Ω^−1^ cm^−2^)	*n*	*R*_ct_ (Ω cm^−2^)	*η* (%)
Control	13.6	73.9	0.74	2189	389.3	0.69	10 196	—
0.0001 M	16.7	21.01	0.68	5842	145.8	0.78	22 165	54.0
0.0005 M	16.2	20.8	0.73	6421	137.3	0.79	25 490	60.0
0.001 M	18.3	19.4	0.76	6387	117.1	0.82	32 890	69.0
0.005 M	15.4	18.8	0.81	7218	102.4	0.85	37 763	73.0
0.01 M	17.1	17.3	0.84	7742	43.5	0.88	53 663	81.0
0.05 M	14.4	16.7	0.88	10 617	38.7	0.89	59 976	83.0
0.1 M	18.3	22.4	0.83	8015	168.9	0.82	37 763	73.0
0.15 M	16.2	24.7	0.77	66.21	183.18	0.73	27 557	63.0
0.2 M	18.9	23.9	0.75	6731	178.4	0.77	29 131	65.0

The low-frequency and high-frequency semi-circles in the Nyquist plot are attributed to the interfacial processes on the steel surface and the passive layer, respectively. The results show that the addition of EDTA resulted in a larger diameter of the capacitive loops in the Nyquist plot. The total impedance of the uninhibited system was more than one order of magnitude lower than that observed in the presence of the inhibitor with the lowest concentration. This behaviour was expected given that the solution was highly contaminated with aggressive ions. As can be seen, the low-frequency impedance of the control sample dropped drastically, showing that the corrosive species reached the steel surface and caused degradation of the passive layer. Both the low-frequency and high-frequency loops were affected by the addition of the inhibitor, where among the concentrations, the low-frequency impedance value showed the highest value for 0.01 M and 0.05 M. The significant difference between the impedance values indicates the effectiveness of the inhibitor in corrosion protection. Theoretically, the intersection points between the Bode phase curve and Bode modulus will move toward the upper left area as the anticorrosive performance increases.^24^ However, although after the addition of the inhibitor, the intersection point moved in the expected direction, the trend of inhibition performance for different contents is controversial. This behavior is attributed to the physicochemical interactions of the organic molecule, which are addressed below.

The most important parameters extracted from the EIS spectra are reported in Table 1. The obtained values for *R*_ct_ increased with an increase in the inhibitor concentration. The inhibitor concentration plays the main role in improving the protective properties of the passive film. The inhibition efficiency of the inhibitor for corrosion was calculated using the charge transfer resistance, as follows:10
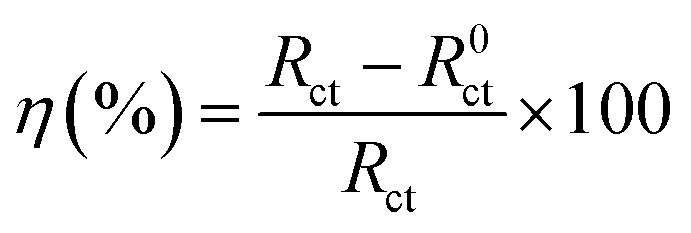
where *R*^0^_ct_ and *R*_ct_ are the charge transfer resistance values in the absence and presence of the inhibitor, respectively. The double-layer capacitance is correlated with the maximum frequency at which the imaginary component (*Z*˙˙) of the impedance is maximum, with the following equation:11
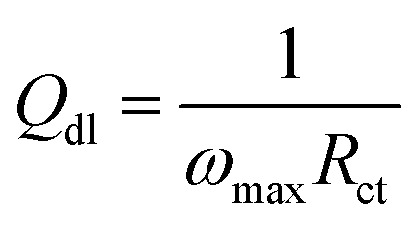


As can be seen in Table 1, the overall response of the system with an increase in the inhibitor content is the improvement in protective properties. The addition of EDTA increased both *R*_ct_ and *R*_p_, which means according to eqn (10), better inhibition efficiency was achieved. Compared to the control sample, the inhibition performance was enhanced at higher contents, and the *R*_ct_ reached value 59 976 Ω cm^−2^ in 0.05 M EDTA with 83% efficiency with respect to the uninhibited sample.

The trend of double-layer capacitance was also favourable given that the *Q*_dl_ value decreased in the presence of the carboxylate compound, which indicates a barrier effect as a result of the resistive behaviour of the system. The lower *Q* value is an excellent indicator of the inhibition performance. The values of double-layer capacitance could also be explained by the Helmholtz model according to the following equation,12
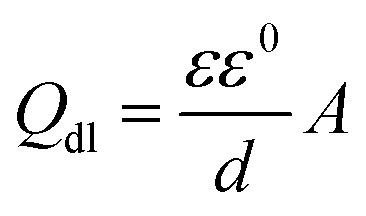
where *A* is the exposed area of the metal surface, *d* is the thickness of the electric double layer, *ε*^0^ is the permittivity of the vacuum, and *ε* is the local dielectric constant. Although the double layer does not comprise ideal capacitance, the decrease in its value can be attributed to either the replacement of H_2_O and OH^−^ by the inhibitor molecule, leading to a thicker electric double-layer and lower local dielectric constant or a reduction in the exposed electrode surface as the result of the adsorption of carboxylate compounds, which both confirm the inhibition performance of the inhibitor.

Due to the frequency-dependent nature of the EIS method, a wide-range analysis of frequency allows the behaviour of the system to be studied in the smallest possible intervals. This is important given that even a simple reaction such as the release of iron as the cation on the metal surface is a combination of various reactions and each of these reactions has their own rate constant, which can only be evaluated through frequency differentiation. Regarding the fact that the capacitance responds only when a charge transfer phenomenon is active and this type of phenomenon is only visible when the time is fragmented *via* frequency (which is the case in the EIS measurement), lower capacitive behavior indicates a lower frequency response, and consequently less active reactions. According to Table 1, an increase in the EDTA content resulted in a decrease in the *Q*_p_ and *Q*_dl_ values with respect to the control sample, which demonstrates the less active surface in the presence of the inhibitor. The parameter *n*, which shows the ideality of the surface heterogeneity, reached almost 0.9, indicating that despite the type of action mechanism, in the presence of carboxylate groups, the surface becomes more uniform.

All the data from the EIS measurement shows the significant effect of EDTA as a corrosion inhibitor in an alkaline environment contaminated with NaCl. However, the efficiency of this organic inhibitor is not entirely consistent with the trend of its content. As can be seen, the increase in concentration was only favorable until it reached 0.05 M. Subsequently, the efficiency moved in the opposite direction and the inhibitive performance started to fail. Eventually, the addition of 0.2 M EDTA resulted in 65% efficiency, which is close to that for 0.001 M. The cause of this behavior is discussed in the adsorption isotherm and quantum calculation sections. However, from an electrochemical point of view, we suggest that there may be three reasons responsible for this type of behavior.

The first is attributed to the polar property of water molecules. Although there is no net charge in a water molecule, the polarity of water creates a slightly positive charge on hydrogen and a slightly negative charge on oxygen, contributing to the properties of attraction of water. The intermolecular forces between the slightly positively charged ends of water molecules to the negative end of another agent such as Cl^−^ or COO^−^ results in the full coverage of negatively charged molecules with H_2_O. Consequently, the mobility of these agents is be affected, where the attractive forces on the surface have to overcome a more voluminous molecule. Given that these agents are simultaneously attracted and covered by more than one water molecule, they are now less mobile with less affinity to move. However, Cl^−^ does not experience the same limitations as COO^−^ given that it has a smaller size, stronger electronegativity, and higher presence in the solution (0.6 M). Although this effect is present at any content of the inhibitor, at very high concentrations, a larger proportion of the electrolyte is involved in the coverage of H_2_O molecules. Therefore, the carboxylate molecules become less available and less mobile to move towards the surface.

The second reason for the efficiency drop at concentrations higher than 0.05 M is ascribed to the ion-pairing effect. As the concentration of various species present in the solution increases, the possibility for ions with opposite charge to contact each other increases. In this situation, some of the anions or cations that once were a neutral compound together, as the result of attractive forces, may move towards each other and become a pair again. For example, it is possible for an Na^+^ ion to be attracted to any anion in the solution, including chlorine or carboxylate ions. Although the saturation point and solubility product constant are involved in this case (which dismisses the case for an anion such as Cl^−^), it is important to note that ion-pairing does not necessarily have to happen in a complete way. We suggest that when the concentration of EDTA exceeds a certain amount, partial pairing with cations in the solution may happen. This results in relatively stable organic anions with less electrical charge, and consequently less affinity for reaching the surface.

The third reason is the carboxylate–carboxylate and carboxylate–surface interactions, which were well explained in the PDM section together with the inhibition mechanism. However, from an electrochemical point of view, we can say that the near-surface carboxylates may contribute to a series of interactions, which would have a repulsive effect towards other species. Without delving into the inhibition mechanism, considering the fact that some of the carboxylate groups on the alkyl chain may not become neutralized with an external positive charge, we suggest that as a result of higher contents of EDTA, the accumulation of carboxylates near the surface would have two effects. The first is that although a large amount of carboxylate molecules inhibits Cl^−^ from coming near the surface, they also have a repulsive effect against other COO^−^ ions in the solution, stopping them from their inhibition action. Another outcome is that the same molecules that have now completed their task of corrosion inhibition, may even have a repulsive effect against each other. In this case, the heterogeneity of the surface is damaged, which may lead to some of the carboxylate anions leaving the surface, providing the opportunity for Cl^−^ to reach the metal. These interactions finally lead to chaos in the electrical double layer, which directly affects the electrochemical behavior of the electrode.

Based on the reasons discussed above, it is reasonable to conclude that at a certain point, the surface can be expected to have the best condition for the adsorbed layer in terms of order and overall coverage. Subsequently, the slightest change in the concentration of either the inhibitor or Cl^−^ has a significant effect on the resulting data given that from this point, the compounds in the solution will perturb the integrity of the adsorbed layer. This is the reason for the considerable change in the efficiency above the optimum concentration.

### 3.3. Potentiodynamic measurement

**Fig. 3:**
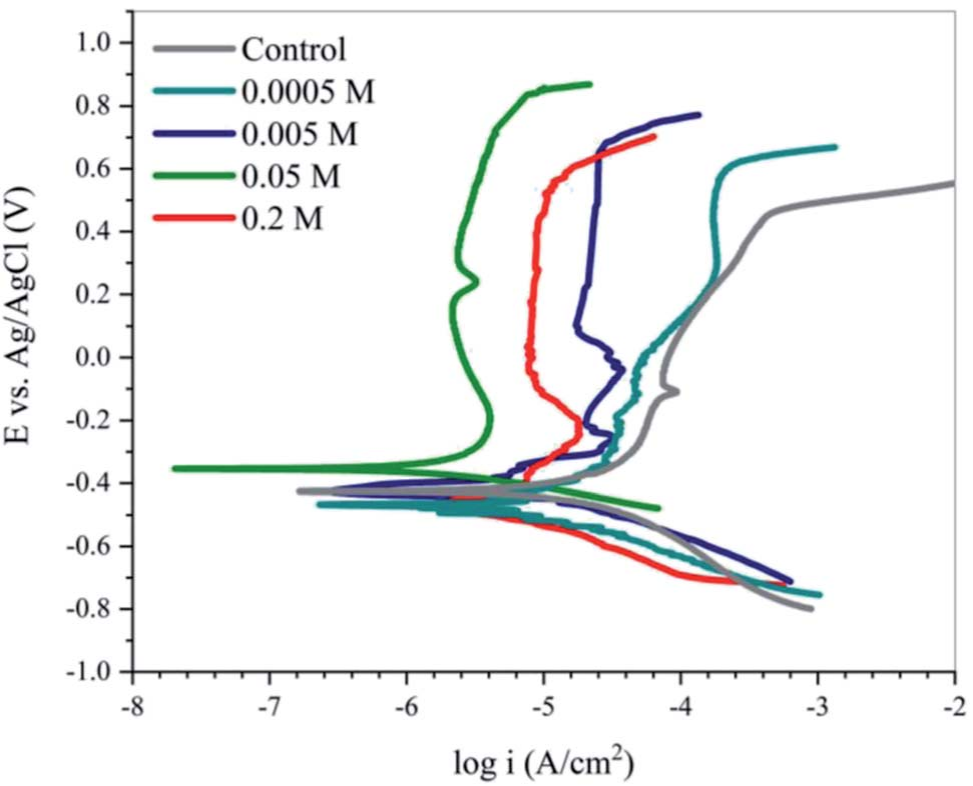
Potentiodynamic polarization curves for C-steel corrosion in SCPS contaminated with 0.6 M NaCl in the absence and presence of various concentrations of EDTA at 25 °C.

To further investigate the corrosion inhibition ability of EDTA, polarization measurements were carried out. The resulting data are and extracted parameters are presented in Fig. 3 and Table 2, respectively. Regardless of the applied content of the inhibitor, a specific drop in both anodic and cathodic current densities occurred, showing that the inhibitor is capable of minimizing the reactions on both the anodic and cathodic sites.^25^ It is clear from Fig. 3 that the addition of inhibitor to the aggressive medium causes a significant reduction in the corrosion rate of the steel samples.

**Table 2:** Corrosion parameters obtained from potentiodynamic polarization measurements for C-steel in SCPS contaminated with 0.6 M NaCl in the absence and presence of various concentrations of EDTA at 25 °C

Sample	*E*_corr_ *vs.* Ag/AgCl (mV)	*I*_corr_ (μA cm^−2)^	*β*_a_ (mV dec^−1^)	*β*_c_ (mV dec^−1^)	CR (mpy)	IE%
Control	−430	12.48	0.235	−0.141	5.69	—
0.0005 M	−455	5.08	0.157	−0.131	2.32	60
0.005 M	−383	3.37	0.113	−0.124	1.54	73
0.05 M	−368	1.87	0.379	−0.059	0.85	85
0.2 M	−477	2.97	0.238	−0.109	1.36	76

It is well known that a ±85 mV change in the *E*_corr_ relative to the potential of the corrosion of the control sample indicates an anodic or cathodic-type inhibitor, regarding its inhibition mechanism. As can be seen in Table 2, the *E*_corr_ for different concentrations of the inhibitor varied within this range, showing that the molecules operate as a mixed-type inhibitor. The inhibition efficiency in the presence of different concentrations of EDTA was calculated using the following equation:13
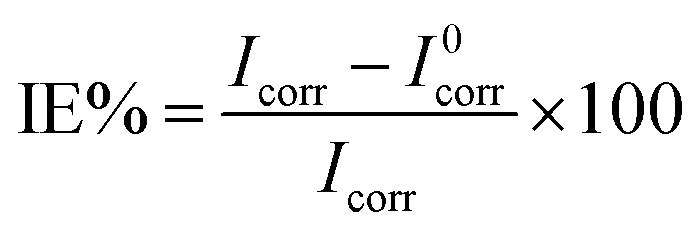
where *I*_corr_ and *I*^0^_corr_ are the corrosion current densities of the steel samples in the simulated concrete pore solution, with and without inhibitor, respectively. The obtained data from the Tafel extrapolation shows that in the presence of the EDTA, the corrosion current density decreased, indicating the ability of carboxylate groups to adsorb on the active sites of the surface. Therefore, the overall response of the system to the addition of inhibitor was an increase in the inhibition efficiency, which is due to the less active reactions on the surface. As it was discovered in the impedance analysis, an increase in the concentration of the inhibitor resulted in an improvement in the IE% values. However, the trend of inhibitor content was not completely consistent with the corrosion inhibition efficiency, given that increasing the concentration to values more than 0.05 M led to lower IE%, where at 0.2 M content, the steel sample showed 61% corrosion inhibition efficiency against the aggressive medium. As mentioned before, the general assumption about the inhibition mechanism of carboxylate-based organic compounds is blocking the active sites of the metal and forming a physical barrier layer by adsorption on the surface. However, it has been reported that there is a possibility that the inhibitor molecules interact with other surface species such as cations of iron or OH^−^ or H_3_O^+^, which may help them in the process of adsorption through a synergetic effect.^26^ This could be the reason for the slight change in the anodic slope (*β*_a_) of the polarization curves for the inhibited samples, given that, as is known, the scanning range for the anodic branch comprises the potentials in which OH^−^ is more stable than before. The results obtained by the potentiodynamic method were in good agreement with the impedance analysis, proving the inhibition effect of EDTA against a corrosive medium contaminated with Cl^−^.

According to the electrochemical measurement results, the passive film was enhanced due to the inhibitive effect of carboxylates on the metal surface as a result of either displacement of chloride ions or the repulsive effect against Cl^−^ during and after the process of adsorption, which is caused by the negative charge on the carboxylate groups. This type of protective film isolates the metal from chloride ions and other aggressive species in the media. The adsorbed molecules with a negatively charged functionality or lone pair electrons develop a repulsive action towards chloride ions, preventing them from coming in contact with the passive layer of carbon steel. Alternatively, alkyl chains or massive functional groups form a type of physical barrier, which blocks chloride contact or delays its approach near the metal surface. These statements are further discussed in the following regarding the quantum chemical calculations. However, before discussing this issue, there should be a good understanding of the formation and degradation mechanism of the passive layer.

### 3.4. Point defect model

It has been reported that^5^ in the competition between anions, the anion with stronger electronegativity and smaller size has the advantage for higher mobility toward the surface. Hence, if the Cl^−^/OH^−^ ratio exceeds a critical amount, chloride ions become adsorbed on the surface and start the degradation of the protective layer. However, this assumption does not fully coincide with the experimental observations, given that whether chlorine was added at the beginning together with Ca(OH)_2_ or after a certain time, at least the local passive state of the surface was achieved, and even in the worst possible conditions, it is just locally damaged and not completely degraded. Therefore, it can be concluded that in an alkaline environment, OH^−^ ions have a better chance of adsorption than any other anion, and a passive layer is formed after a very short amount of time. However, when the passive state is accomplished, in the absence of the inhibitor, if the Cl^−^ content exceeds a critical value, due to the parameters mentioned above and also the alteration in the nature of the surface, the adsorption of chlorine is favored more than OH^−^. Before discussing the inhibition performance and the action mechanism of EDTA, the functionality of the passive layer should be addressed.

As mentioned earlier, the passive film has an Fe_3−*δ*_O_4_ (0 < *δ* < 0.33) structure, the stoichiometry of which changes with the thickness, where the end number of the series is maghemite, γ-Fe_2_O_3_ (*δ* = 0.33). Maghemite has a bilayer spinel structure, consisting of an inner layer of Fe_3_O_4_ and an outer layer of γ-Fe_2_O_3_ or its hydrated form FeOOH.^27^ Both layers are formed electrochemically and are defective with n-type semi-conductive properties.^28^ It has been reported that the anion vacancies that are formed at the Fe/Fe_3−*δ*_O_4_ interface and move to the oxide surface become occupied by Cl^−^, leading to the formation of FeCl_2_.^29^ This product either precipitates on the surface and forms a non-protective porous layer or dissolves in the solution, which in the second case, once again, Cl^−^ returns to the system, and repeats the cycle.^30^ It should be noted that even under steady-state conditions, corrosion and passivation coexist simultaneously. Thus, before addressing corrosion inhibition, it is crucial to discuss the passivation phenomena. For this purpose, with the help of other research on this subject, a brief version of the point defect model was used to identify the very first interactions leading to corrosion inhibition.

**Fig. 4:**
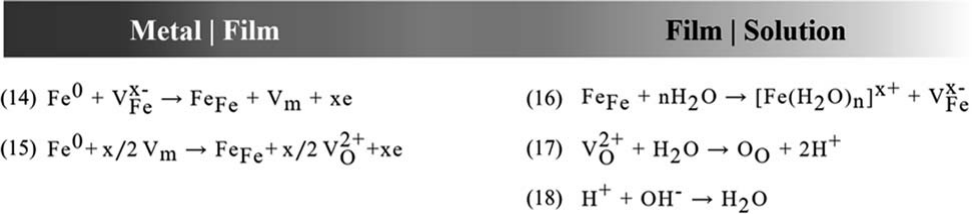
Summary of generation I PDM reactions for the formation and annihilation of point defects that occur at the interface affecting barrier oxide layer on the metal.

The PDM is a theoretical model for explaining the formation and breakdown of the passive film on metals and alloys.^31–34^Fig. 4 shows the reaction scheme proposed by the PDM for metal/film and film/solution interface. According to theory, the oxide growth results from a series of processes that point defects such as cation V^*x*−^_M_ and anion vacancies, V^2+^_O_ are involved in, which lead to a defective layer of MO_*x*/2_.^35^ Based on the PDM, the breakdown of passivity induced by anions, *e.g.*, Cl, has been attributed to the occupation of the near-surface oxygen vacancies,^29,33^

14 *V*^2+^_O_ + 2X^−^ → 2X_O_

This reaction is the very first interaction that results in unstable ferrous compounds. Furthermore, it results in a thermodynamically unstable condition for the passive layer given that the anion vacancies are now out of balance. Therefore, it is followed by a Schottky-pair reaction,

15 Null → *V*_M_ + (*x*/2)*V*_O_

Adsorption on anion vacancies leads to the generation of cation vacancies at the film/solution interface. According to eqn (19), the enhanced flux of cation vacancies through the film leads to their accumulation at the metal/film interface. Once this null area grows to a critical size, dissolution of the film at the film/solution interface and tensile stresses in the barrier layer induce mechanical, chemical, and structural instability, resulting in passivity breakdown.^36^

According to the PDM, the general process of pit formation can be described as (i) dissolution of aggressive ions into oxygen vacancies, (ii) generation of cation vacancies at the film/solution interface, (iii) diffusion of cation vacancies through the oxide layer and their subsequent annihilation by metal oxidation at the metal/film interface, and (iv) the cation vacancies that are not annihilated completely condense to form voids, resulting in local film detachment and the onset of pit initiation.^9^ In this model, an important feature is the autocatalytic and simultaneous generation of oxygen and cation vacancies at the film/solution interface.

**Fig. 5:**
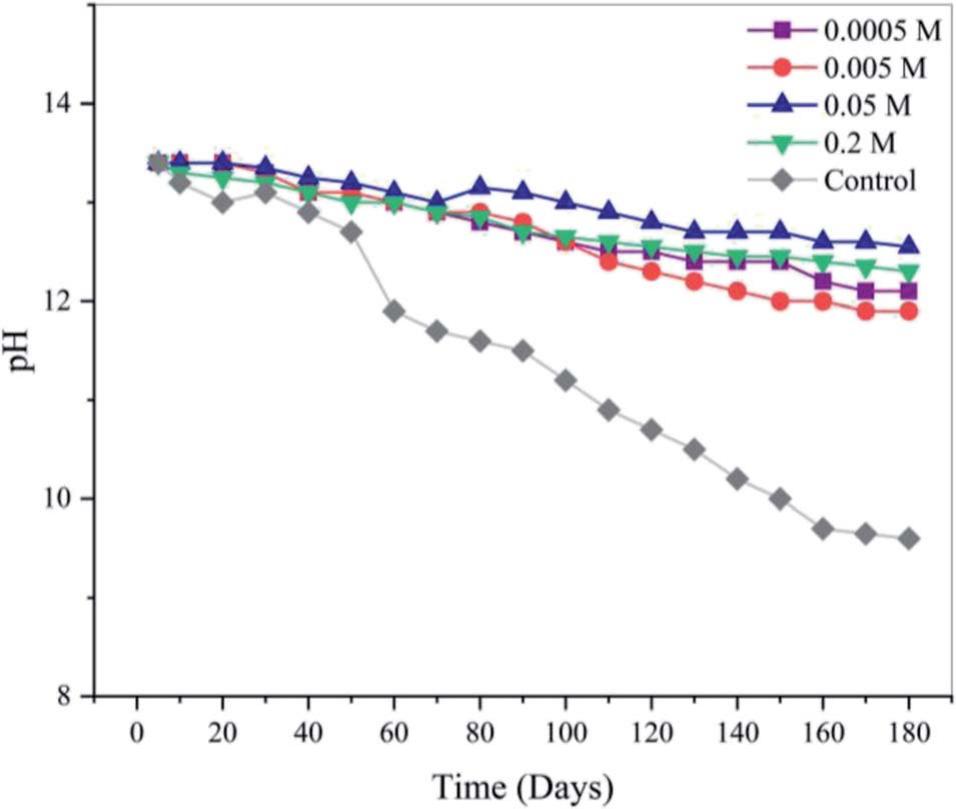
pH change in uninhibited and inhibited SCPS in a 6 month period.

According to Fig. 4 and eqn (18), the surface adsorption of water results in the formation of H^+^ ions. Fig. 5 shows the pH change in the uninhibited and inhibited SCPS in a 6 month period. Due to the highly alkaline nature of the solution, H^+^ ions formed on the surface and became neutralized with the hydroxides, but after a certain amount of time, according to Fig. 5, not only the alkalinity of the solution changed, but the localized pH on the film surface also decreased drastically. It was observed that the pH of the solution almost reached the value 9 after 6 months of exposure.

As can be seen, anion vacancies play a significant role in the adsorption of negatively charged ions and decrease in the localized pH at the surface. However, the vacancies are not the only entities involved in passivation processes. The reaction scheme presented in Fig. 4, which is based on generation I PDM, does not include metal interstitials as one of the point defects. The generation II PDM introduced metal interstitials to the suite of defects and recognized the dissolution of the barrier layer. Other generations of PDM have also been developed recently to either extend the theory to special cases such as valve metals, which have an extremely resistive outer layer (PDM-III), or to describe the passivity on alloys (PDM-IV). Among the interpretations of lattice theory, PDM-II has achieved considerable success given that it is applicable for a variety of cases and has shown no instance of being inconsistent with the experimental results.

According to recent studies,^37^ in an n-type oxide, there must be a mobile donor other than oxygen vacancies; therefore, the existence of cation interstitial defects should be considered. Eqn (21) and (22) show the reactions involved in the generation and annihilation of interstitials, respectively;

16 Fe^Fe^ → Fe^*x*+^_i_ + *V*_m_ + *xe*

17 Fe^*x*+^_i_ → Fe^*n*+^_(aq)_ + (*n* − *x*)*xe*

where Fe^*x*+^_i_ and Fe^*n*+^_(aq)_ are the cation interstitial and cation released at the outer layer/solution interface, respectively. This additional assumption (together with other postulates that we do not wish to discuss herein) was necessary to achieve an accurate description of the physicochemical processes occurring at the interface. As mentioned earlier, the oxide film on the steel is a bi-layer structure of an inner layer Fe_3_O_4_ known as the barrier layer, which has a more condensed structure and an outer layer, commonly comprised of an oxide, oxyhydroxide, or hydroxide (or a mixture of these species). The barrier layer contains only the species that are present in the substrate metal and not that existing in the solution. Therefore, we can conclude that cation interstitials are first formed in the barrier layer. Then, due to their high mobility, after entering the outer layer of the film, they may reach the surface and be released as cations at the film/solution interface. These cations may interact with other anion species in the solution and form ferrous compounds. This presumption coincides with the experimental data obtained by other researchers,^30,38,39^ proving the existence of ferrous complexes other than common oxides on the surface. However, the significant role of cation interstitials at the film/solution interface is the occupation of cation vacancies as a result of the Schottky-pair reaction (eqn (20)). This is very important given that because of this event, the destructive effect of oxygen vacancies leading to the formation of cation vacancies is partially be neutralized. Therefore, based on a more developed form of the PDM, we should consider both anion vacancies and cation interstitials as the dominant entities affecting the state of passivity.

Although oxygen vacancies are the main origin of passivity breakdown and the degradation of the film, they also are the source of action in the inhibition mechanism. We suggest that the inhibition mechanism of carboxylate molecules is through their adsorption on anion vacancies together with the release of cations at the outer layer interface. As mentioned before, there are four carboxylate groups (two on each end) on an EDTA molecule with a delocalized negative charge on the two electronegative oxygen atoms. According to quantum chemical calculations,^11^ the charge of a carboxylate group is approximately equally distributed over both its oxygens, which means this molecule acts as a single negatively charged unit. Given that there are two dominant positively charged entities in the passive film, which are the near-surface oxygen vacancies and released cations at the interface, and considering the fact that an EDTA molecule comprises four COO^−^ groups, it is reasonable to assume that immediately after the exposure, each molecule would experience a powerful attractive force. It should be mentioned that the other negatively charged species in the solution such as Cl^−^ and OH^−^ would experience the same attractive force from the oxide defects, which causes them to compete with each other to reach the surface.

We suggest that the competitive action mostly favors the carboxylates by physical and chemical adsorption simultaneously. Physical adsorption is attributed to the oxygen vacancies, whereas the metal cations on the surfaces are responsible for chemical adsorption. Because of the spatial shape of the molecule and the two carboxylates on the tail, there may be a repulsive effect against other aggressive anions. In addition, chemisorption most likely leads to the formation of some surface complexes, which help to create a protective barrier along the surface. These hypotheses are further discussed in the following section with the help of adsorption isotherms, computer simulation, and surface analysis techniques.

In the case of the relevance of this notion to the electrochemical parameters, the defective structure of a passive oxide defines both the nature and the performance of the electrical double layer. The double-layer is described as two parallel layers of charge, consisting of a first layer, where the ions are chemically adsorbed on the surface, and a second layer, which is attracted to the surface *via* the coulombic forces from the first layer. According to Helmholtz's theory,^40^ the two layers of opposite polarity formed at the electrode/electrolyte interface are essentially the components of a molecular dielectric, which stores charge electrostatically. Based on the point defect model, we can conclude that the charge and the composition of the first layer are directly dependent on the type of mobile defects in the passive film, such as vacancies and interstitials. In the case of the pH environment, Macdonald *et al.* proved that the type of these entities are oxygen vacancies and metal interstitials.^33^ Therefore, the nature of the DL would be a first layer of negatively charged species such as COO^−^ or OH^−^ and a second layer of solvated ions that compete to reach the surface, either to attack or to replace the first layer. The performance of the DL, and consequently the electrochemical behavior of the sample are also dependent on the point defects given that there the number of active defects that can interact with the solution is limited. This is the main reason for the reduction in the inhibition efficiency at concentrations above 0.05 M EDTA, given that there are no more active mobile defects near the surface to be used by the excessive carboxylate groups as adsorption sites.

### 3.5. Adsorption isotherms

Physical and chemical adsorption are two main types of adsorption of organic inhibitors on a metal surface. As was demonstrated by the PDM, corrosion inhibition of organic compounds is directly correlated with the adsorption phenomenon, which is evaluated through different adsorption isotherms.

**Fig. 6:**
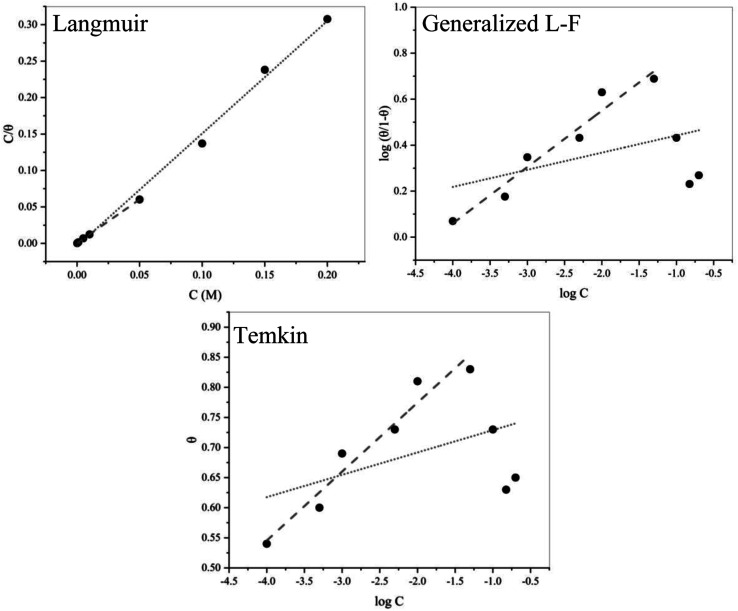
Partial (⋯) and full (---) fitting of the Langmuir, generalized L–F and Temkin adsorption isotherms for EDTA.

The adsorption isotherm plots and calculated parameters for EDTA are presented in Fig. 6 and Table 3, respectively. The equilibrium constant for the adsorption reaction, *K*_ads_, is related to the standard free energy for adsorption, Δ*G*_ads_, by the following equation:^41^18
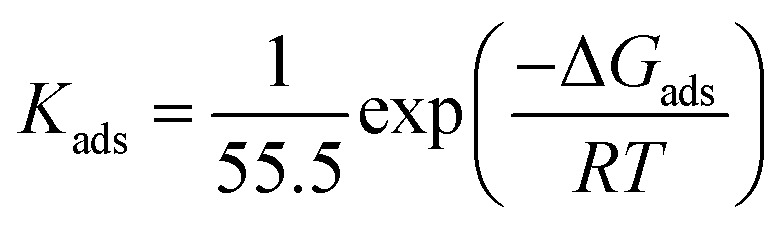
where *T* is the absolute temperature and *R* is the ideal gas constant.

**Table 3:** Fitting results for the Langmuir, generalized L–F and Temkin isotherms for EDTA

		*h*	*f*	*K*_ads_ (10^3^ × L mol^−1^)	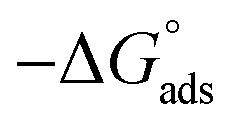 (kJ mol^−1^)	*R* ^2^
Langmuir	Partial	—	—	2.93	29.7	0.998
	Full^a^	—	—	8.33	32.3	0.995
Generalized L–F	Partial	0.25	—	17.6	34.1	0.94
	Full	0.075	—	8547.15	49.5	0.19
Temkin	Partial	—	20.1	579998.4	59.9	0.95
	Full	—	62.1	4.4 × 10^17^	127.7	0.21

a The error bar was taken to account.

As mentioned before, the performance of the inhibitor is not entirely consistent with the trend of its concentration given that the increase in concentration was only favourable until it reached 0.05 M. Based on the thermodynamic nature of adsorption, the variation in the performance of EDTA at concentrations higher and lower than 0.05 M could be investigated in thermodynamic terms. In this study, three isotherms were used to evaluate the adsorption behaviour based on their underlying assumptions. To scrutinize the variation in performance at higher contents of EDTA, a partial fit of the isotherms was also applied for 0.0001–0.05 M EDTA, which provides valuable information on the important factors that lead to the reduction in efficiency from a thermodynamic point of view.

According to Table 3, the data has the best fit with the Langmuir isotherm. The Langmuir isotherm is based on series of assumptions, which can be summarized as (i) all the adsorption sites are equivalent, and each of them can only be interlocked with one molecule, (ii) the surface is energetically homogeneous, and there is no interaction between the adsorbed molecules, (iii) no phase transitions will occur, and (iv) at the maximum possible adsorption, only a monolayer is formed. However, these assumptions are rarely all true because there are always imperfections on the surface, adsorbed species are not necessarily static, and the process is clearly not the same for the very first molecules to adsorb on a surface as the last. Nonetheless, comparing the degree of linearity of the three isotherms as measured by values of *R*^2^, it can be seen that the Langmuir adsorption isotherm is the most applicable for this case.

The negative values of 
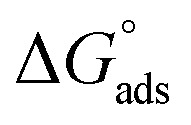
 suggest that the inhibitor is spontaneously adsorbed on the surface. The adsorption processes are generally classified as physisorption (characteristic of weak van der Waals forces) or chemisorption (characteristic of covalent bonding).^42^ With values of 
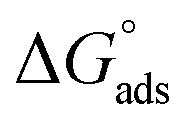
 of up to −20 kJ mol^−1^, the mechanism of adsorption is physisorption, while chemisorption happens when this value is around −40 kJ mol^−1^ or higher.^43,44^ The 
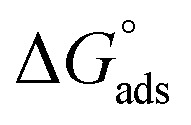
 value for the Langmuir model was −32 kJ mol^−1^, which implies a mixed mechanism of simultaneous physical and chemical adsorption. As discussed earlier in the PDM section, the physical adsorption of EDTA is assumed to occur as a result of electrostatic attraction by the near-surface oxygen vacancies, while the chemisorption of carboxylate molecules occurs due to chelation on the surface after the electron transfer to the unoccupied d-orbitals of the released metal cations.^5^

Although the Langmuir isotherm gave the best fit, the 0.94 and 0.95 values obtained for the regression coefficient of the partial fit for Temkin and generalized L–F, respectively, show the reliability of the results obtained for contents less than 0.05 M. Therefore, we can presume that the assumptions on which these isotherms are based can also be applied here. The Temkin adsorption model is believed to account for some of the factors not considered in the Langmuir model, such as molecular interactions in the adsorbed layer. The magnitude of the molecular interaction parameter is related to the coulombic repulsion forces experienced by the inhibitor molecules during adsorption as a result of neighboring adsorbed or adsorbing species. In the case of long-chained or voluminous molecules, molecular crowding or steric effects can also have an effect on the *f*-values, which is the case for EDTA. Comparing the value of 20.1 for the molecular interaction constant with that for other carboxylate compounds obtained by fitting the Temkin adsorption isotherm by other researchers,^45^ a good understanding of the heterogeneities in the adsorbed layer of EDTA molecules on the steel surface can be achieved. We suggest that although the Langmuir model best fits the obtained results, because of its oversimplified assumptions, it fails to present an accurate prediction of the interactions in the adsorbed layer. Hence, we believe that the repulsive forces between the inhibitor molecules intensify at a higher concentration, leading to a localized nonintegrated imperfect multi-layer of adsorbed molecules with weakened corrosion inhibition properties, which no longer obeys the generalized L–F or Temkin isotherms. This assumption is consistent with the results from the EIS and potentiodynamic measurements.

Another important reason that proves the dysfunctionality of the Langmuir model is the fact that it does not incorporate the possibility of the formation of surface complexes. As mentioned before, EDTA is a strong complexing agent, which is capable of complexing ferric ions. There are two nitrogen atoms with a free electron pair and four acidic hydrogens on each EDTA molecule. The (EDTA)^4−^ anion can occupy four, five or six coordination sites around a central metal cation.^46^ The central charge can be provided by a metal interstitial that has reached the surface. It can also be provided from separate Fe^2+^ and Fe^3+^ ions, which results in the formation of ferric EDTA. The principal formula for this complex is [Fe(EDTA)(H_2_O)]^−^, which is either going to be electrostatically adsorbed by the surface or react with other metallic ions such as Na^+^ to form Na[FeEDTA(H_2_O)]·2H_2_O or Na_4_[(FeEDTA)_2_O]·3H_2_O.^47^

It should be mentioned that the degree of complex formation by EDTA depends on the pH value of the environment. According to the literature,^48^ Fe–EDTA remains largely stable at low pH, but at pH higher than 8, the solubility of iron drops to about 5% after 3 days and further decreases rapidly with time. Although the characterization techniques in that study cannot be considered as absolutely reliable, this notion is still factual. Therefore, despite the fact that the chelation effect of EDTA is one of the mechanisms responsible for corrosion inhibition, it cannot be considered as the most effective one.

Whether the surface is blocked by ferric EDTA or protected by surface adsorption, the Langmuir isotherm cannot provide any coherency with the actual inhibition mechanism. Therefore, we believe that the use of the Temkin and generalized L–F is essential for addressing the metal/electrolyte interactions.

### 3.6. Quantum chemical calculations

**Fig. 7:**
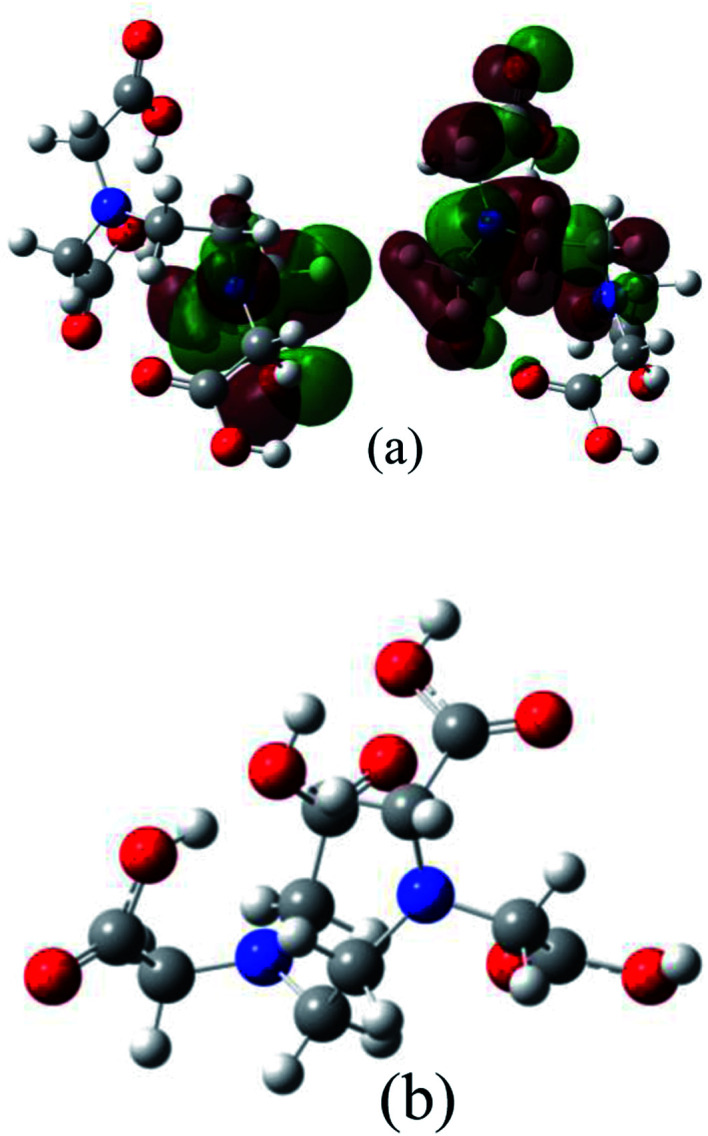
Illustration of (a) HOMO and LUMO and (b) optimized geometry configuration of EDTA obtained from quantum chemical calculations.

Quantum chemical studies on a compound can help to understand the surface interactions and the corrosion performance of an inhibitor molecule. The calculated quantum chemical parameters are reported in Table 4. An illustration of the molecular structures of EDTA and the frontier molecule orbital density distributions are shown in Fig. 7.

**Table 4:** Quantum chemical parameters for EDTA

	*E*_HOMO_ (eV)	*E*_LUMO_ (eV)	Δ*E* (eV)	Dipole moment (*D*)	*I* (eV)	*A* (eV)	*χ* (eV)	*η* (eV)	*S* (eV^−1^)	Δ*N*
EDTA	−6.09	−1.72	4.37	4.79	6.09	1.72	3.90	2.18	1.46	0.71
TCNE	−11.09	−9.78	1.31	0	11.09	9.78	10.44	0.657	1.52	−2.61

It is well known that the frontier molecular orbitals are indicators of the chemical reactivity of organic compounds given that based on molecular orbital theory, the transition state at the time of reaction is induced by the distribution of the frontier orbitals of reactants.^49,50^ In addition, the energy of the highest occupied and lowest unoccupied molecular orbitals reflects the molecular interaction with the surface, which may or may not result in the prevention of corrosion. The HOMO and LUMO are known for their electron donor and accepter ability, respectively. A high value of *E*_HOMO_ indicates the tendency of molecules to donate electrons to unstable entities on the metal surface, therefore contributing to corrosion inhibition. Alternatively, the lower the value of *E*_LUMO_, the higher its ability to accept electrons. It should be noted that although the donation ability of a negatively charged molecule such as carboxylate is crucial, the back donation of an electron from a metal or other entities on the surface is also very important. Therefore, the energy gap (Δ*E*) between the orbitals as a function of reactivity was used to appraise the inhibition properties of EDTA on the corroding surface. The energy gap is a stability index, where a large value of Δ*E* is indicative of a stable compound in a chemical reaction. It is well known that good corrosion inhibitors have low energy gap values given that the reactivity of the molecule increases as Δ*E* decreases. Compared to TCNE as the reference molecule,^18^ EDTA shows approximately the same reactivity and less electron-accepting ability, which reflects how this molecule interacts with the metal by donating its electrons in its HOMO, leading to blocking the active sites on the surface. Fig. 7b shows the molecular structure of EDTA. In the optimized geometry, it can be seen that there is a single carboxylate group, oriented in line with the axis of the alkylene chain, which is separate and away from the other parts of the molecule. We suggest that this specific group has the greatest contribution to the inhibition action, and even in the case of possible multi-group adsorption on the surface, this particular COO^−^ group would be the first to participate in the inhibition mechanism. The illustration of the HOMO in Fig. 7a also supports this statement given that the electron distribution of the HOMO is widely spread over this carboxylate group.

The dipole moment (*D*), which is an indication of the polarizability of a molecule in the presence of electrostatic forces, can play a role in the adsorption of the inhibitor. The spatial orientation of a molecule depends on its polarity. It has been reported that a high dipole moment value enhances the mobility of the inhibitor towards the metal, and consequently leads to electrostatic adsorption on the surface. As can be seen in Fig. 7a, there is a strong distribution of electron density over the HOMO on the upper part of the alkylene chain, suggesting the orientation of the molecule in the presence of an electrostatic force. This results in a specific position of the molecule on the surface, which suggests that the mechanism can even be through the interaction between the π-electron lone pair of the N atoms and vacant d-orbital of the metal atoms.^51^

Another important parameter exported from the quantum analysis is the hardness and chemical softness. It has been reported that a lower value of *η* increases the inhibitor ability towards the deformation or polarization of the electron cloud when it is experiencing a small perturbation at the time of chemical reaction.^52^ Regarding the fact that the adsorption on the surface happens at the parts of the molecule that have a greater global softness, EDTA is expected to have good corrosion inhibition. Electron-donating ability, which in some reports is recognized as the number of electrons transferred from the inhibitor to metal, was also derived from quantum chemical calculations.^53,54^ It has been reported that if Δ*N* > 0, the electrons are transferred from molecule to the metal, which in this case are transferred from the inhibitor to free d-orbital of iron. This confirms the proposed mechanism by the PDM, showing that in addition to physical adsorption, the inhibition action can be through chemisorption by forming a covalent bond with the cation species on the surface.

### 3.7. Field emission scanning electron microscopy

**Fig. 8:**
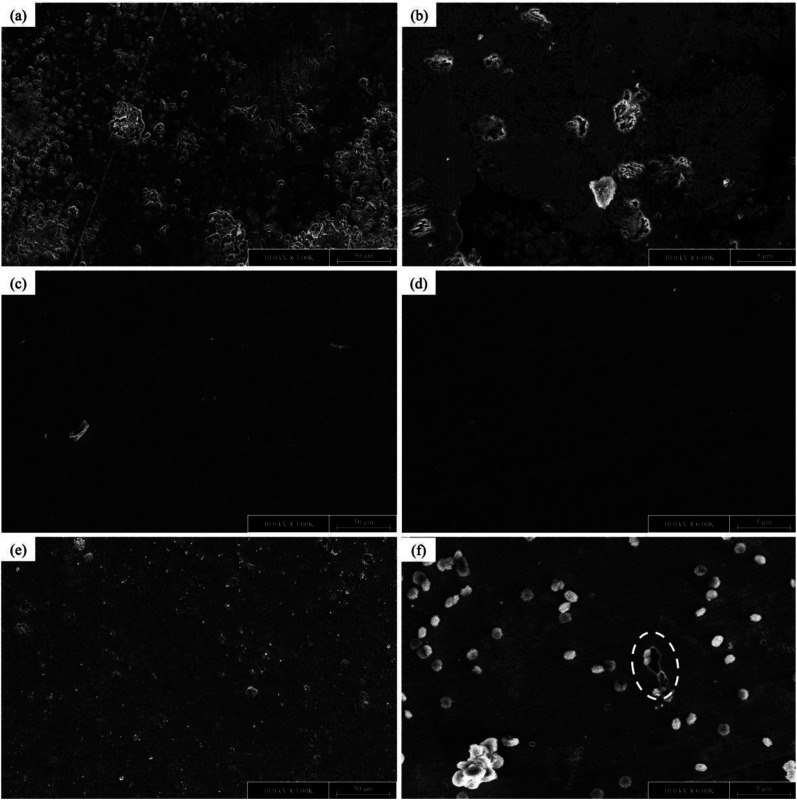
FE-SEM images of C-steel surface in SCPS contaminated with 0.6 M NaCl in the absence (a and b) and presence of 0.05 M (c and d) and 0.2 M (e and f) corrosion inhibitor, after 48 hours of exposure.

Fig. 8 shows the microstructure and morphology of the surface, which were obtained for the 0.05 M, 0.2 M and inhibitor-free samples. According to Fig. 8a, in the absence of EDTA, localized corrosion of the surface was observed. This is obviously because of the presence of chloride ions, which attacked the surface, leading to the formation of non-compact and porous corrosion products. The highly alkaline nature of the solution led to the formation of a strong protective passive film on the surface as an obstacle against aggressive agents. However, as mentioned in the PDM section, the entities on the passive layer forced Cl^−^ to move towards the surface, leading to the formation of unstable products. Because these products are highly porous with electrochemical properties different from that of the passive film, they were recognized as new active sites for aggressive ions to attack. Hence, a severe form of localized corrosion occurred. Eventually, after a certain amount of time, these phenomena resulted in catastrophic corrosion pits.

In the presence of 0.05 M and 0.2 M EDTA, the morphology changed. As can be seen in Fig. 8c and d, the surface is more homogenous, and no sign of elevations can be observed in the control sample. This is due to the uniform adsorption of the inhibitor on the surface. The sample with 0.2 M EDTA also showed a relatively smooth surface; however, some localized pits were observed, which may have been formed because of the perturbation of the adsorbed layer as a result of either excessive negatively charged groups in the solution or the disturbance caused by the intermolecular repulsion within the adsorbed layer. Some accumulation of complexes was also observed, showing the chemical reactions between the carboxylate groups and metal cations, leading to insoluble chemical compounds. Although the microstructure of the sample exposed to 0.2 M EDTA was less homogeneous than that exposed to 0.05 M, the overall observation is that in the presence of EDTA, the adsorbed layer on the passive film resulted in a smoother and plainer surface with an improved barrier effect. This finding is in agreement with the results from the electrochemical measurements.

### 3.8. X-ray photoelectron spectroscopy

**Fig. 9:**
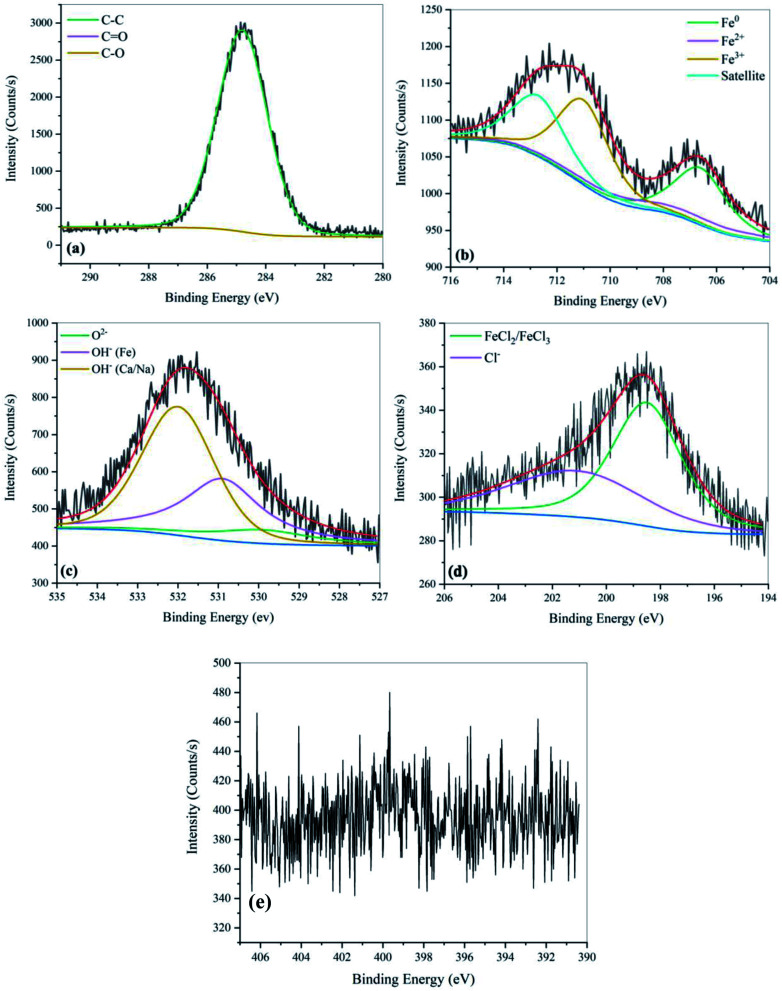
XPS spectra of carbon steel after 48 hours of immersion in SCPS with 0.6 M NaCl. (a) C 1s, (b) Fe 2p_3/2_, (c) O 1s, (d) Cl 2p, and (e) N 1s.

**Fig. 10:**
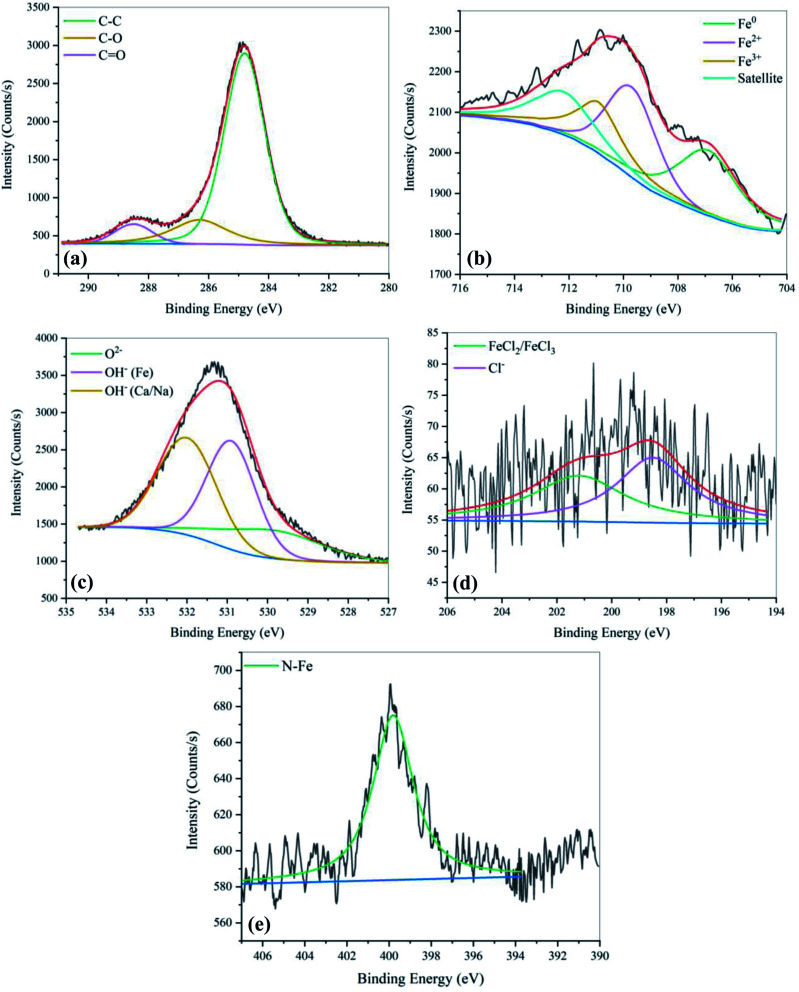
XPS spectra of carbon steel after 48 hours of immersion in SCPS with 0.6 M NaCl in the presence of 0.05 M EDTA. (a) C 1s, (b) Fe 2p_3/2_, (c) O 1s, (d) Cl 2p, and (e) N 1s.

The metal surface was also studied through the X-ray photoelectron spectroscopy technique to investigate the composition of existing elements and chemical bonds. The XPS spectra for the inhibitor-free and 0.05 M inhibited samples are shown in Fig. 9 and 10, respectively.

The XPS high-resolution spectra of C 1s for the sample with the addition of 0.05 M EDTA was split into three different peaks at 288.7 eV, 286.4 eV,^55^ and 284.8 eV,^56^ corresponding to the C=O, C–O and C–C bonds, respectively. As can be seen, the C–O and C=O bonds were not observed for the inhibitor-free sample, which is attributed to the absence of carboxylate groups on the surface. It is worth mentioning that in some studies, the simultaneous presence of a single and double bond of carbon–oxygen in the XPS spectra is considered as an index for COO^−^ groups,^57^ which further confirms the adsorption of the inhibitor on the surface. The pattern for Fe 2p_3/2_ of the passive layer on the steel specimens displays that the surface is mostly comprised of Fe^3+^ (711 eV)^39^, Fe^2+^ (708 eV)^58^ and Fe^0^ (706.6 eV)^59^. As is known, the maximum depth of analysis for an XPS instrument is 10 nm. Hence, the higher intensity of Fe^0^ peak observed for the uninhibited sample shows a non-uniform oxide layer with lower thickness than that with inhibitor. In addition, the presence of Fe^0^ on the surface can be considered as an indicator of the rates by which the reversible reactions of corrosion occurred, showing a less stable passive state. Based on the literature, the broad peaks centered at 707.6 eV and 712.7 eV can be attributed to Fe_3_O_4_ and Fe_2_O_3_ or its hydrated forms, respectively.^46,58,60^ To further discuss the protective properties of the passive layer, the XPS parameters were exported from the plots and are presented in Table 5.

**Table 5:** Variations in passive film composition in the absence and presence of EDTA

Sample	Fe^0^ (%)	Fe^2+^ (%)	Fe^3+^ (%)	Satellite (%)	Fe^2+^/Fe^3+^ (%)	O^2−^ (%)	OH^−^ (%)	Ca/Na OH^−^ (%)	O^2−^/OH^−^ (%)
Control	24.4	13.2	26.6	35.8	0.49	10.68	46.93	42.39	22.75
EDTA-0.05 M	20.6	28.7	20.3	30.4	1.41	20.23	39.48	40.29	51.24

As mentioned in the PDM section, the spinel structure of the passive film consists of an outer layer of γ-Fe_2_O_3_ and an inner layer of Fe_3_O_4_, which is more condensed with a better barrier effect. According to Table 5, with the addition of EDTA, Fe^2+^, which is responsible for the formation of Fe_3_O_4_, increased, while a slight decrease in Fe^3+^ was observed. This phenomenon can be attributed to the fact that with the passive layer becoming more stable, the inner layer starts growing into the second layer, which causes the transformation of Fe(iii) to Fe(ii).^61^ The amount of O^2−^ also increased as it became double the initial amount after the addition of the inhibitor. Both the Fe^2+/^Fe^3+^ and O^2−^/OH^−^ ratios increased in the presence of 0.05 M EDTA, which demonstrates the higher stability and better protective property of the passive layer.

**Fig. 11:**
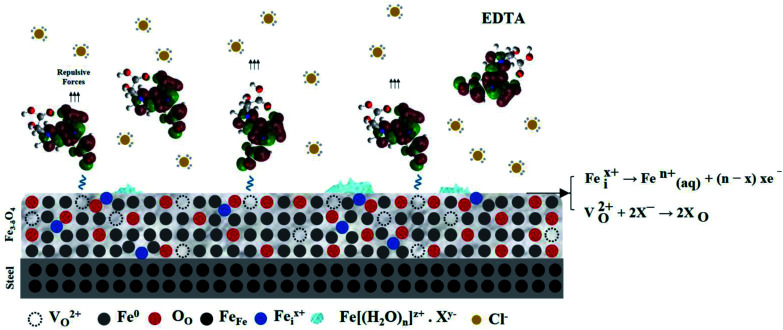
Illustration of the corrosion inhibition mechanism of EDTA on the defective structure of the passive layer based on the point defect model.

As illustrated in Fig. 10c and 11c, the O 1s signal is asymmetric and shows three distinct peaks at 529.9 eV, 530.9 eV and 532 eV.^62^ The first signal corresponds to the O^2−^ in iron oxide, the intensity of which can be attributed to the level of passive oxide film entanglement on the surface. The other two peaks correspond to the metallic hydroxides derived from the surface and environment. According to these figures, the addition of EDTA to the SCPS enhanced the iron hydroxide peak, but the peak attributed to the iron oxide remained almost unchanged. This can be related to the fact that after the addition of the inhibitor, not only the outer layer became more uniform (according to the stable and equal distribution of iron hydroxide based on the value of peak densities), but the passive layer became thicker, exceeding the detection range of the XPS instrument.

The N 1s spectra show the N–Fe bond at a binding energy of 399.8 eV.^63^ This confirms the chemical adsorption of the EDTA molecules on the surface. The Cl 2p spectra were also analyzed to investigate the protective and repulsive effect of the inhibited sample against chloride ions. The Cl on the surface was mainly presented as peaks at 198.5 eV and 201.1 eV, which are due to Cl^−^^64^ and FeCl_2_/FeCl_3_, respectively.^65^ As illustrated in the Fig. 10d and 11d, the decreased intensity of chloride ions shows that the organic molecules successfully hindered the adsorption of Cl^−^ on the surface. This confirms the corrosion inhibition property of EDTA.

A brief illustration of the inhibition mechanism is shown in Fig. 11, which is the comprehensive understanding of the authors from the interfacial interactions between carboxylate-based organic compounds and metal substrate.

## 4. Conclusion

Electrochemical measurements proved that at 0.05 M EDTA, the best corrosion inhibition performance was achieved in carbon steel. The results showed that before reaching the optimum concentration, the trend for the protection level of the metal against aggressive medium coincided with the increment in the content of the inhibitor. However, with the further addition of the compound to the system, the inhibition efficiency started to decrease because of the medium polarity, ion-pairing effect and carboxylate–carboxylate and carboxylate–surface interactions.

Due to the defective nature of the oxide film on the metal, the point defect model was employed as a tool for investigating the surface interactions. We suggested that after the carboxylates are pulled to the surface as a result of electrostatic forces, physical and chemical adsorption simultaneously occur. This fact was experimentally proven by the adsorption isotherms, where the values of 
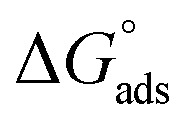
 showed mixed-type adsorption. It was suggested that the physical adsorption is attributed to the anion vacancies, which play a very important role in the mobility of the carboxylate groups towards the surface. However, the metal cations on the surface are considered to be responsible for chemical adsorption, which is a more stable form of protection. In addition, it was proven that the presence of EDTA molecules hinders the localized pH drop at the surface, and hence reduces the possibility of the formation of metastable pits. It was also confirmed through XPS analysis that in the presence of the inhibitor in the system, Cl^−^ and its derivatives were less observed on the surface, while the C–O, C=O and N–Fe peaks, which are representative of carboxylate compounds, were identified clearly, confirming the adsorption of the inhibitor. Despite the good correlation of the data with the Langmuir isotherm, physical or chemical adsorption of the inhibitor would not necessarily happen entirely based on its assumptions, and thus more inclusive isotherms such as the Temkin adsorption isotherm can offer a more precise prediction of what of the mechanism on the surface. We believe that the molecular crowding and steric effects caused by the long-chained or voluminous molecules resulted in repulsive forces between the free species on the surface. This effect intensified at a higher concentration, leading to a localized nonintegrated imperfect multi-layer of adsorbed molecules with weakened corrosion inhibition properties.

Based on the quantum chemical calculations, the specific distribution of the electron density over the HOMOs on the upper part of the alkylene chain suggests the particular position of the molecule on the surface at the time of adsorption. This is important in two ways. Firstly, based on the distribution of carboxylate groups on the chain and the shape of the molecule, the way that the molecules relax on the surface defines the level of repulsive forces against aggressive agents such as Cl^−^ ions. Secondly, if the concentration exceeds a certain amount, due to the same reason, the repulsive effect can expand along the adsorbed layer, which results in a non-uniform layer with less protective properties. This was also observed in the SEM images, where the surface became less smooth and more heterogeneous when the concentration of carboxylate inhibitor increased to 0.2 M.

## Conflicts of interest

There are no conflicts to declare.

## Supplementary Material
